# Suspended animation-inducer hydrogen sulphide protects against organ injury during endotoxemia, but aggravates systemic inflammation

**DOI:** 10.1186/cc9622

**Published:** 2011-03-11

**Authors:** H Aslami, C Beurskens, F De Beer, M Kuipers, M Schultz, N Juffermans

**Affiliations:** 1 Academic Medical Centre, Amsterdam, the Netherlands

## Introduction

A suspended animation-like state induced by hydrogen sulphide (H_2_S) was shown before to protect lungs from ventilator-induced lung injury by reducing metabolism and inflammation. This beneficial effect of H_2_S seems promising, but the effects of H_2_S during prolonged infusion are unknown. We hypothesized that reducing metabolism in a rat model of LPS-induced systemic inflammation during 8 hours is more protective than during 4 hours.

## Methods

After anesthesia, rats (400 g) received an intravenous injection with 7.5 ml/kg LPS and were subsequently randomized to 4 or 8 hours of mechanical ventilation and treated with intravenous H_2_S donor NaHS (2 mg/kg/hour). Controls received saline. During the experiment, mean arterial pressure (MAP) was kept above 65 mmHg with fluids and noradrenalin infusion. After exsanguination, bronchoalveolar lavage fluid was obtained and organs were harvested. Data are mean ± SEM.

## Results

H_2_S reduced metabolism, exemplified by a reduction in heart rate, body temperature and etCO_2 _compared with saline controls. Also, oxygenation was improved in these groups. The H_2_S-treated animals required more noradrenalin to keep the MAP above 65 mmHg. LPS-induced lung injury was reduced after 4 hours of H_2_S infusion compared with controls, with lower BALF protein levels (399 ± 46 vs. 655 ± 85 μg/ml), IL-6 levels (4.5 ± 0.3 vs. 6.2 ± 0.6 ng/ml) and CINC3 levels (2.4 ± 0.09 vs. 2.9 ± 0.2 ng/ml) (*P *< 0.05 for all), whereas 8 hours of infusion did not enhance protection. Kidney injury, measured by wet-to-dry ratio, was reduced after 8 hours of H_2_S infusion compared with saline controls (5.5 ± 0.1 vs. 6.1 ± 0.1 ratio, *P *< 0.05). The cumulative fluid balance was the same in all groups. In contrast to the protective effect at tissue level, H_2_S infusion resulted in enhanced systemic levels of IL-1, IL-6, TNF and CINC3 compared with saline controls.

## Conclusions

During endotoxemia, 4 hours of H_2_S infusion protected against lung injury, which was not further enhanced by 8 hours of infusion. In contrast, kidney damage was diminished after 8 hours but not after 4 hours of H_2_S infusion. However, H_2_S aggravated systemic inflammation in endotoxemia, suggesting that administration of H_2_S gas may be preferable.

